# THE RELATIONSHIP BETWEEN ADVANCE DIRECTIVE COMPLETION AND POTENTIALLY AVOIDABLE HOSPITALIZATIONS

**DOI:** 10.1093/geroni/igz038.1310

**Published:** 2019-11-08

**Authors:** Colleen M Galambos, Marilyn Rantz, Lori Popejoy, Pritchett Angelita, Greg Petroski

**Affiliations:** 1 University of Wisconsin Milwaukee, Milwaukee, Wisconsin, United States; 2 University of Missouri Sinclair School of Nursing, Columbia, Missouri, United States; 3 Office of Medical research, Columbia, Missouri, United States

## Abstract

Advance directive (AD) completion can improve transitions between hospitals and skilled nursing facilities (SNF’s). One CMS Innovations Demonstration Project, The Missouri Quality Initiative (MOQI), focused on improving advance directive documentation and use in sixteen SNF’s. An analysis was conducted of data collected from annual chart inventories occurring over four years. Using a logistic mixed model, results indicated statistical significance (p<0.001) for increased AD documentation. Greatest gains occurred at project mid-point. The relationship between having an advance directive and occurrence of transfer to a hospital was tested on a sample of 1563 residents with length of stays more than 30 days. Residents who did not have an advance directive were more likely to be transferred. A logistic regression was conducted and the results were statistically significant (p<0.02). The MOQI model and initiatives will be explained followed by a discussion of research methodology, data collection, and analyses. Practice implications will be discussed.

